# A pilot study of rifle and head kinematics during 0.50 caliber rifle firing

**DOI:** 10.3389/fbioe.2026.1907502

**Published:** 2026-09-29

**Authors:** Luděk Hynčík, Tomasz Bońkowski, Aleš Franc, Martin Kučera, Arya Nabavi, Karol Miller, Michal Soták, Jan Páleník, David Netuka, Martin Májovský

**Affiliations:** 1 Department of Biomechanical Human Body Models, New Technologies - Research Centre, University of West Bohemia in Pilsen, Pilsen, Czechia; 2 Intelligent Systems for Medicine Laboratory, School of Engineering, The University of Western Australia, Perth, WA, Australia; 3 Department of Infrared Technologies, New Technologies - Research Centre, University of West Bohemia in Pilsen, Pilsen, Czechia; 4 Department of Neurosurgery, Medizinische Hochschule Hannover, Hannover, Germany; 5 Department of Anesthesiology and Intensive Care, First Faculty of Medicine, Charles University and Military University Hospital, Prague, Czechia; 6 Department of Neurosurgery and Neurooncology, First Faculty of Medicine, Charles University and Military University Hospital, Praha, Czechia

**Keywords:** firing, head, high-caliber rifle, kinematics, muzzle break

## Abstract

**Introduction:**

This study analysed rifle and head kinematics during firing, using automated motion tracking to quantify maximum rifle displacement, head displacement, and head acceleration.

**Methods:**

Six professional snipers participated in repeated firing trials under controlled conditions. Additional trials were performed for selected participants to show recoil conditions without a muzzle brake.

**Results:**

Rifle displacement ranged from 36.30 to 67.65 mm and head displacement from 25.86 to 44.55 mm with the muzzle break, whereas without the muzzle brake values increased substantially to 76.36–102.97 mm and 76.47–82.47 mm, respectively. Peak head acceleration showed a less consistent pattern, ranging from 3.71 to 8.62 g with the muzzle brake and 5.10–8.71 g without it. Interindividual variability was observed across all measures, suggesting an influence of participant-specific biomechanical and anthropometric characteristics on recoil transmission.

**Discussion:**

The findings from this pilot study indicate that the use of a muzzlebrake may reduce the translational recoil motion of both the rifle and theshooter, whereas peak head acceleration appears to be less affected. However, as the condition without a muzzle break was evaluated in only two participants with a limited number of trials, these observations should be considered preliminary and descriptive rather than conclusive. The results also suggest that individual biomechanical factors may influence recoil response, highlighting the need for larger, balanced studies to better understand the effects of muzzle brakes and their potential implications for ergonomics and shooting performance optimisation.

## Introduction

Rifle discharge has increasingly been recognized in military biomechanics and occupational exposure research not only as a ballistic event, but as a complex human-rifle interaction system involving coupled mechanical recoil, blast overpressure, and whole-body vibration. In high-caliber platforms such as 0.50 BMG rifles, these phenomena are substantially amplified due to the large momentum transfer associated with projectile propulsion and the intense muzzle blast generated during firing. As a result, the mechanical and pressure loads transmitted to the shooter differ significantly from those observed in lower-caliber weapon systems, both in magnitude and temporal structure (Lang et al., 2018; Skotak et al., 2019).

Field studies of 0.50 caliber rifle training environments have demonstrated that shooters can be exposed to measurable blast overpressure during repeated firing sequences. These exposures are strongly dependent on firing posture, muzzle brake configuration, and environmental reflections, with peak values reaching levels considered relevant in occupational blast research (Lang et al., 2018). Multi-day training studies further indicate that such exposures are not isolated events but may accumulate over time, leading to repeated low-level blast exposure scenarios in operational settings (Skotak et al., 2019). In parallel, biomarker-based investigations have reported changes in neurotrauma-associated biochemical indicators following repeated exposure to 0.50 caliber rifle blast environments, suggesting potential systemic physiological effects even in the absence of clinically apparent injury (Thangavelu et al., 2020).

Recent advances have extended beyond environmental exposure measurements toward direct quantification of operator biomechanics under recoil conditions. Ouellet and St-Onge (2021) measured head kinematics in snipers firing 0.50 caliber rifles using instrumented and high-fidelity motion tracking approaches. Their findings demonstrated that even highly trained operators exhibit measurable head motion and acceleration during firing events, indicating that recoil is transmitted through the rifle-body interface and contributes to coupled head-rifle dynamics rather than being fully absorbed at the shoulder.

Complementary work has further investigated the physiological consequences of recoil exposure at the level of brain response. Seeburrun et al. (2024) assessed brain-related responses in operators exposed to recoil forces from firing high-caliber rifles and reported measurable neuro-mechanical effects associated with repeated firing. These findings suggest that impulsive recoil loading may not only induce musculoskeletal response but may also contribute to transient biomechanical loading conditions relevant to brain tissue deformation, depending on posture and stabilization strategy.

Despite these advances, the current literature remains fragmented. Existing studies typically focus either on external blast exposure metrics, isolated head kinematics, or physiological/biochemical outcomes, rather than integrating all components into a unified dynamic system. In particular, simultaneous measurement of rifle motion, head kinematics, and transmission characteristics in professional sniper populations remains limited.

From a broader biomechanical perspective, rapid head acceleration and rotational motion are known to be key contributors to brain tissue strain leading to mild traumatic brain injury (mTBI) (Naumenko et al., 2023), such as sports-related impacts and blast exposure research. The severity of mTBI depends on the rotational acceleration and impact direction. The brain twists or shears at an awkward angle inside the skull, tearing neural pathways rather than receiving a straight, direct force. Although firearm recoil differs in origin and duration from these loading conditions, the fundamental mechanical principle of inertial brain response to head acceleration remains relevant and motivates detailed investigation of recoil-induced head dynamics.

While peak head accelerations during recoil of high-caliber rifle (typically <10 g) are orders of magnitude lower than the injury thresholds established for football players (typically <100 g as reported by Rowson and Duma, 2013) or boxing (>100 g as reported by Cournoyer and Hoshizaki, 2019), the primary occupational hazard lies in cumulative exposure. Repetitive micro-trauma transferred through the coupled rifle-shooter system induces deep, sub-concussive brain tissue strain (Ouellet and St-Onge, 2021). Over time, this cumulative mechanical and pressure loading manifests clinically as diminished neurocognitive performance, memory deficits, and delayed reaction times (Thangavelu et al., 2020; Laskowitz et al., 2024).

Overall, the literature converges on three key insights: (i) high-caliber firearm discharge produces measurable mechanical recoil and blast-related exposures (Aare and Kleiven, 2007) and leads to decreasing cognitive capabilities (Hunfalvay et al., 2022); (ii) repeated exposure can induce measurable physiological and biomechanical responses in operators (Woodall et al., 2023); and (iii) there is a significant gap in integrated experimental characterization of the coupled rifle-shooter system, particularly with respect to head kinematics and acceleration transmission during firing (Seeburrun et al., 2024). Addressing this gap is essential for improving understanding of exposure mechanisms and informing ergonomic design, training protocols, and safety standards in high-caliber weapon systems.

## Methods

The study was approved by the Ethics Committee of the Military University Hospital, Prague, Czech Republic, under No. 108/20-38/2025, and the Ethics Committee of the University of West Bohemia in Pilsen under No. V10/2025. The studies were conducted in accordance with the local legislation and institutional requirements. The participants provided their written informed consent to participate in this study. All procedures were designed to protect participant safety, confidentiality, and voluntary participation. All participants were healthy volunteers, and measurements were collected during routine training activities.

The study protocol was prospectively registered in the ClinicalTrials.gov database under No. NCT06992570 and conducted in accordance with ethical principles for human research.

The study included six professional snipers (n = 6), comprising four males and two females, all with extensive operational experience in high-caliber rifle systems. Participants were recruited from trained military personnel and were familiar with standardized firing protocols and recoil management techniques.

All participants first completed the firing condition with the muzzle brake. The condition without the muzzle brake was subsequently performed by two participants only, due to safety considerations associated with the more demanding exposure. The order of conditions was not randomized or counterbalanced. A rest period of several minutes was provided between individual shots.

All participants fired from a prone position directly from the ground, using the same bipod setup and the same bipod height. The rear of the rifle was supported and controlled by the shooter’s hand rather than by a rear bag or other external support.

The firing position was standardized across all participants, including hand placement and cheek contact with the stock. All participants had undergone standardized training in the handling and operation of the rifle and were instructed to follow the same firing technique and recoil management strategy during the experiment. Although parameters such as shoulder preload and cheek contact pressure were not quantitatively measured, participants were required to maintain the standardized firing position and handling technique throughout the testing.

All firing experiments were conducted using a Desert Tech HTI precision rifle chambered in 0.50 BMG (12.7 × 99 mm), see Figure 1. The rifle was equipped with a 29-inch (736.6 mm) barrel with a 1:15 rifling twist rate, a three-chamber muzzle brake, a bipod, and a March 3–24 × 52 riflescope featuring an FML-T1 reticle. The overall length of the weapon was 116.2 cm. The complete firing configuration weighed 11.0 kg with the muzzle brake installed and 10.7 kg after its removal.

**FIGURE 1:**
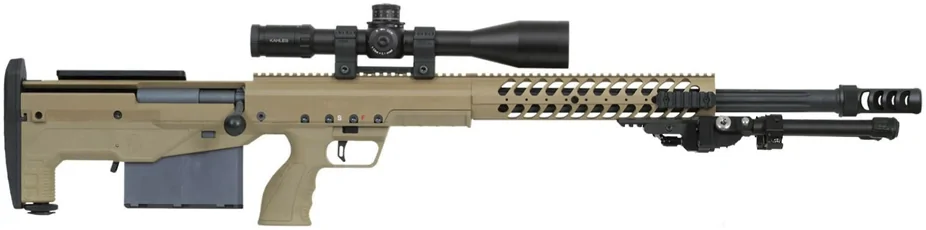
Hti precision heavy caliber rifle 0.50 BMG.

Sellier & Bellot M33 Ball ammunition was used throughout the experiments. The projectile weight was 655 grains, and the manufacturer-specified muzzle velocity was 880 m/s. Muzzle velocity was not independently measured during the experiments. All cartridges originated from the same production lot; however, the lot number was not available. The rifle was supported in the same manner during all shots. When comparing firing conditions with and without the muzzle brake, no component of the firearm, optic, support, or overall configuration was modified other than the removal of the muzzle brake.

Head acceleration was measured using a tri-axial IEPE accelerometer (Kistler 8763B500BB, Kistler Instrumente AG, Winterthur, Switzerland) mounted on the participant’s forehead using a firmly secured adjustable strap and synchronized with a high-speed camera system (Fastec Imaging TS3). The accelerometer has a measurement range of ±500 g, a mass of 4.6 g, and sensitivities of 10.68 mV/g (X-axis), 10.36 mV/g (Y-axis), and 10.73 mV/g (Z-axis) at 159.2 Hz. The frequency response of all three axes remained within ±3% over the 20–10,000 Hz range. Data were acquired using Kistler LabAmp charge amplifiers with a Type 5165A4 data acquisition system (software version 5165AX-V1.3.1-0-gdb599dd; hardware version 550,113) at a sampling frequency of 62.5 kHz. The Type 5165A4 LabAmp automatically applies anti-aliasing filtering for sampling rates ≥25 kSps, with the cut-off frequency set to 0.3–0.45 times the selected output update rate. Accelerometer calibration was performed according to Kistler test procedure 978-5484–702 before testing.

The sensor coordinate system consisted of three orthogonal axes defined relative to the participant’s forehead so that the Z-axis was oriented anteriorly (forward), the X-axis inferiorly (downward), and the Y-axis laterally to the left. Acceleration signals were acquired at 62.5 kHz and subsequently processed in accordance with SAE J211. Signal conditioning was performed using a zero-phase Channel Frequency Class (CFC) 1,000 low-pass filter to avoid phase distortion and temporal shifting of peak values. The tri-axial resultant acceleration was calculated from the filtered orthogonal acceleration components as

$$a_{res}=\sqrt{a_{x}^{2}+a_{y}^{2}+a_{z}^{2}}$$

As the employed IEPE accelerometer does not provide a DC response, static gravitational acceleration was not measured and therefore no gravitational correction was applied. Prior to each measurement, data acquisition commenced approximately 30 s after final positioning of the participant, including accelerometer attachment, rifle handover, adoption of the firing posture, and aiming. This stabilization period minimized motion-related transients, while the use of a portable battery power supply reduced electrical line-noise interference. Consequently, no systematic pre-event offset was observed in the recorded signals, and no additional baseline correction was required.

Peak head acceleration was defined as the maximum value of the tri-axial resultant acceleration within a predefined 10 ms analysis window. Both raw and filtered acceleration-time histories were visually inspected to confirm that the identified peaks corresponded to the primary mechanical loading event and were not attributable to electrical noise, sensor ringing, or resonance artefacts.

Visual tracking targets were placed on both the rifle and the shooters’ head to enable motion tracking and kinematic reconstruction of rifle and head dynamics during firing events, see Figure 2.

**FIGURE 2:**
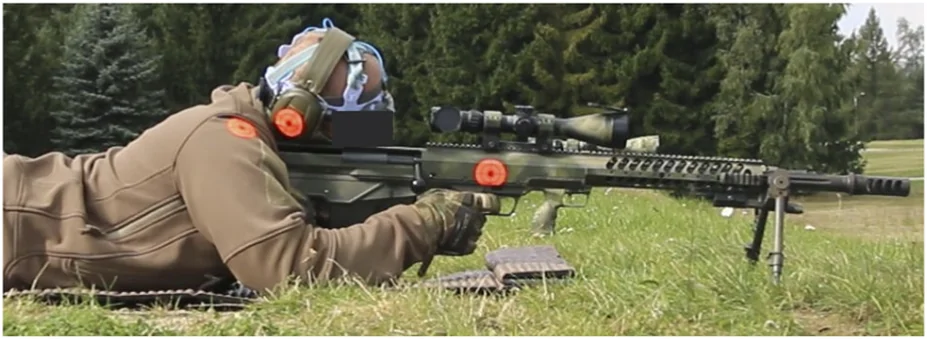
Visible targets.

The experiments were recorded using a FASTEC TS3100 high-speed camera operating at a frame rate of 1,000 frames/s. Images were acquired with a spatial resolution of 888 × 710 pixels using a cropped region of interest positioned at (196, 156) on the sensor. Each recording consisted of 1700 frames, corresponding to a total recording duration of 1.7 s. The camera was configured with an exposure time of approximately 125 μs to minimize motion blur during rapid movement, and the images were stored in JPEG format using 8-bit intensity data.

Red circular markers with black rings and a central dot were used as tracking targets to ensure high visibility in the high-speed camera recordings, see Figure 2. Calibration of the motion recordings acquired with the high-speed camera was based on the known dimensions and shape of the tracking markers placed at the monitored locations. The high-speed camera was positioned at the same height as the subject and levelled horizontally using the spirit level on the tripod. To improve measurement accuracy, the distance between the camera sensor plane and the subject was also measured using a Bosch DLE 70 laser distance meter. The uncertainty associated with this distance measurement was ±3 cm. No perspective correction was applied due to the camera positioning.

Automated tracking of the rifle and head markers was performed using a custom-developed MATLAB algorithm, which processed high-speed camera recordings to extract kinematic trajectories for subsequent analysis of rifle-head motion coupling.

The tracking algorithm was developed in MATLAB using a template-matching approach. For each target, the template was manually defined in the first frame by selecting the marker centre. A template size of 10 × 10 pixels was used, corresponding approximately to the diameter of the marker as captured by the high-speed camera. The template was subsequently tracked throughout the image sequence by maximizing the similarity between the predefined template and the corresponding image region in each frame. The tracking accuracy was evaluated based on the ability of the algorithm to consistently identify the marker centre. The uncertainty of the tracked position was estimated to be within approximately ±1 pixel, corresponding to ±4.47 mm after conversion using the camera calibration factor.

The spatial calibration of the high-speed camera images was performed using a known reference distance. The distance between the rifle head and the centre of the rifle target was measured physically and divided by the corresponding number of pixels in the image, resulting in a pixel size of 4.47 mm/pixel. This calibration factor was determined with the high-speed camera positioned at the same recording distance and orientation as during the firing experiments.

Based on the kinematic data obtained from automated tracking, the maximum displacement vectors of the rifle and head targets were calculated.

## Results

Six professional snipers were analysed (n = 6) using repeated firing trials under controlled rifle recoil conditions. The dataset included rifle displacement, head displacement, and head acceleration for each participant. Table 1 summarizes the anthropometric characteristics of all participants.

**TABLE 1:** Anthropometric characteristics.

Participant (#)	Age (years)	Gender (M/F)	Height (cm)	Weight (kg)
1	35	M	185	82
2	35	M	180	84
3	46	F	171	80
4	38	F	167	68
5	43	M	187	115
6	33	M	183	89

Each participant completed five repeated firing trials under the same experimental conditions, allowing for assessment of within-subject variability in head kinematics and rifle recoil dynamics. Participants #1 and #5 additionally performed firing trials without a muzzle brake, consisting of one additional trial for Participant 1 and two additional trials for Participant #5, allowing exploratory comparison of recoil conditions.

Based on the measured displacements, corresponding displacement vectors were computed (see Figure 3) to characterize the directional motion of both the rifle and the head during recoil events. These derived vectors enabled a more detailed assessment of the spatial coupling and kinematic relationship between the weapon system and the participant.

**FIGURE 3:**
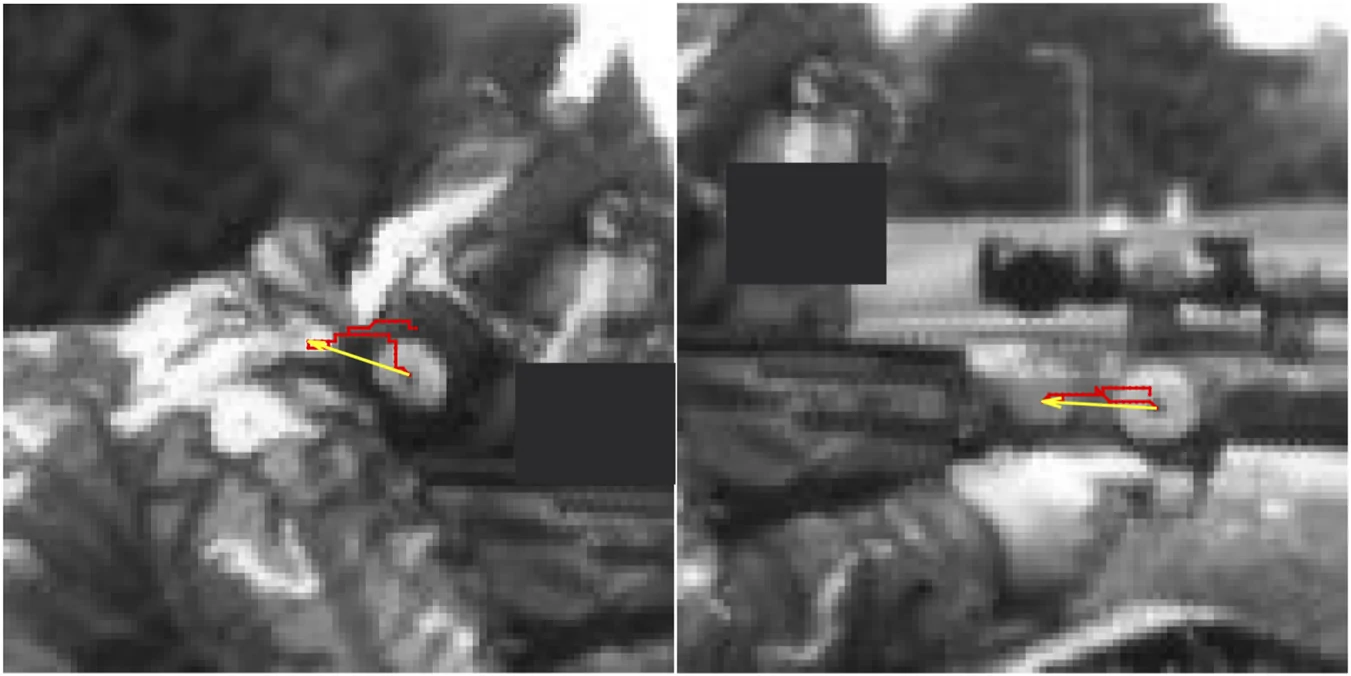
An example (trial 5/2) illustrating how the displacement vectors for head (left) and rifle (right) were calculated by automated tracking.

The displacement vectors for all participants and firing trials are presented in Figures 4–9 for the rifle configuration with the muzzle brake installed and in Figures 10, 11 for the configuration without the muzzle brake.

**FIGURE 4:**
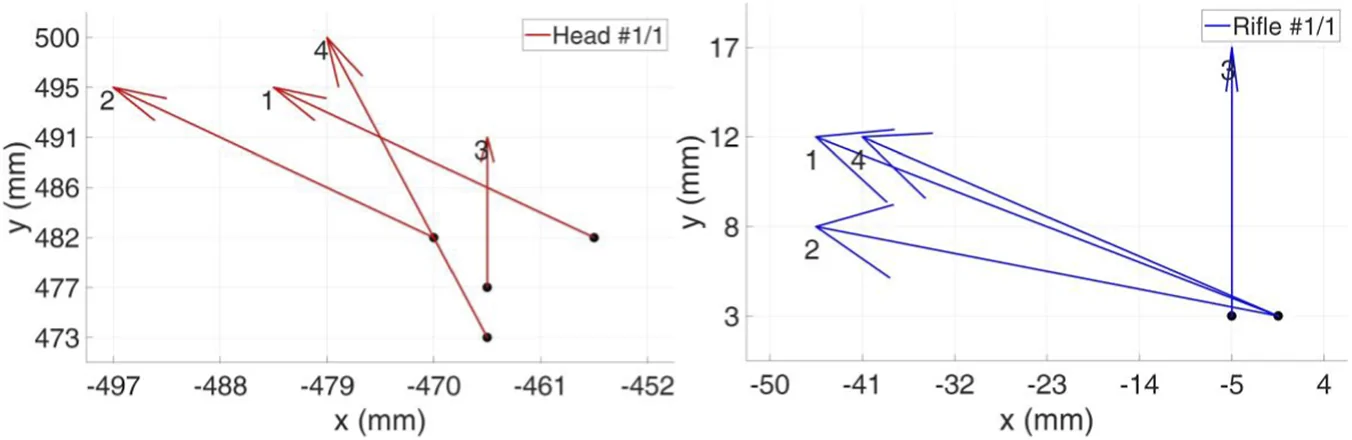
Head (left) and rifle (right) targets displacement for trial #1/1.

**FIGURE 5:**
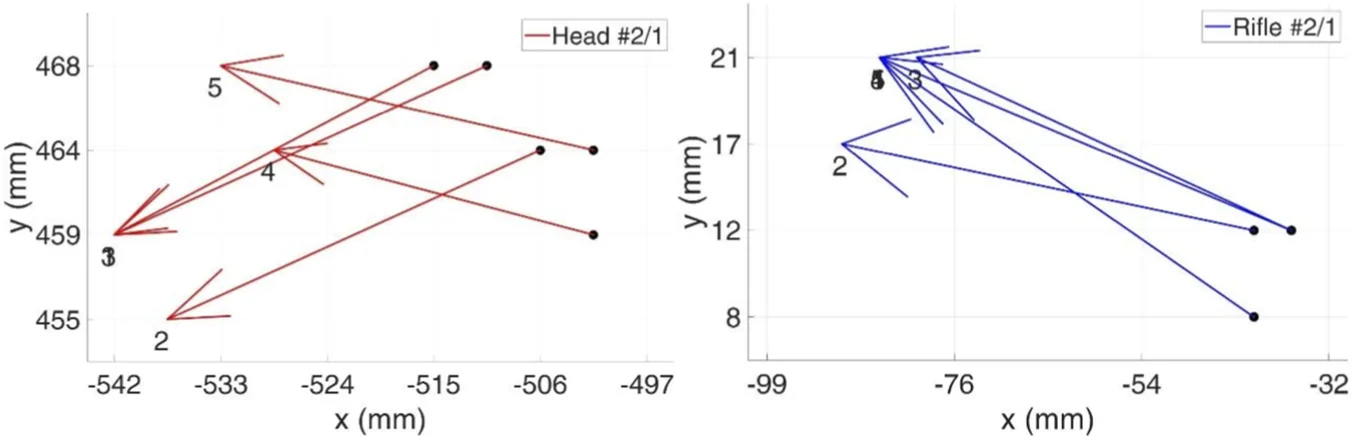
Head (left) and rifle (right) targets displacement for trial 2/1.

**FIGURE 6:**
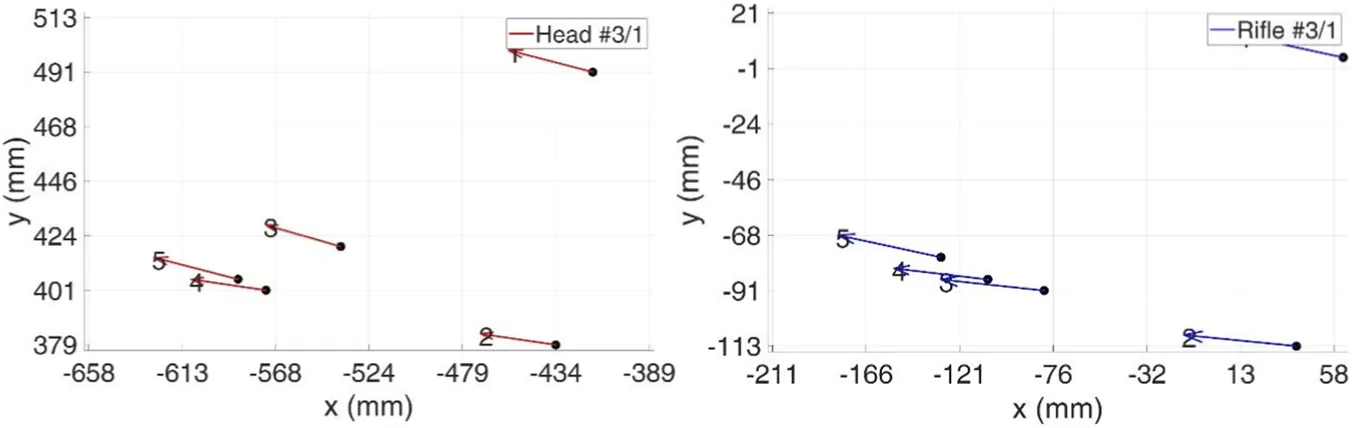
Head (left) and rifle (right) targets displacement for trial 3/1.

**FIGURE 7:**
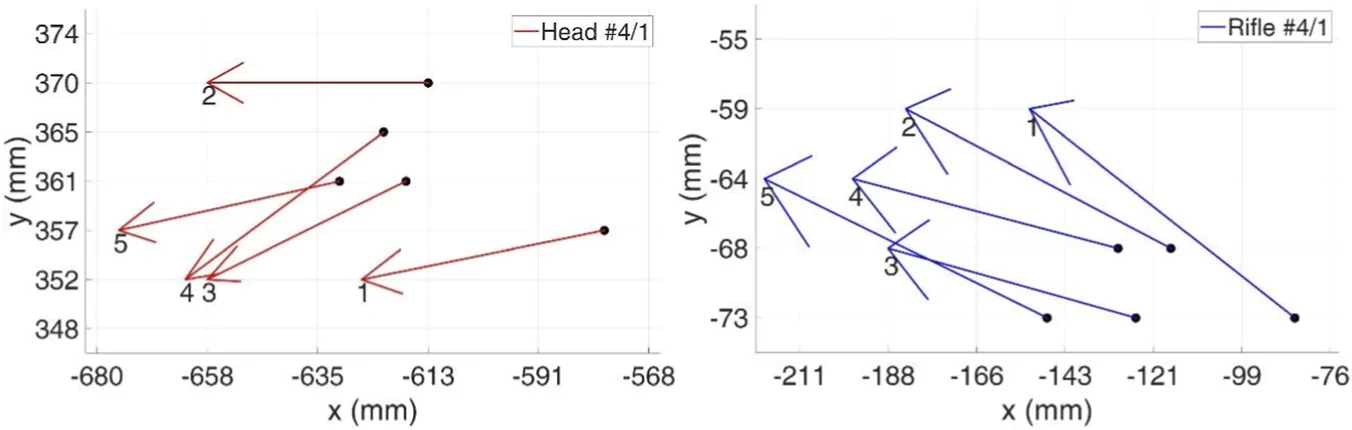
Head (left) and rifle (right) targets displacement for trial 4/1.

**FIGURE 8:**
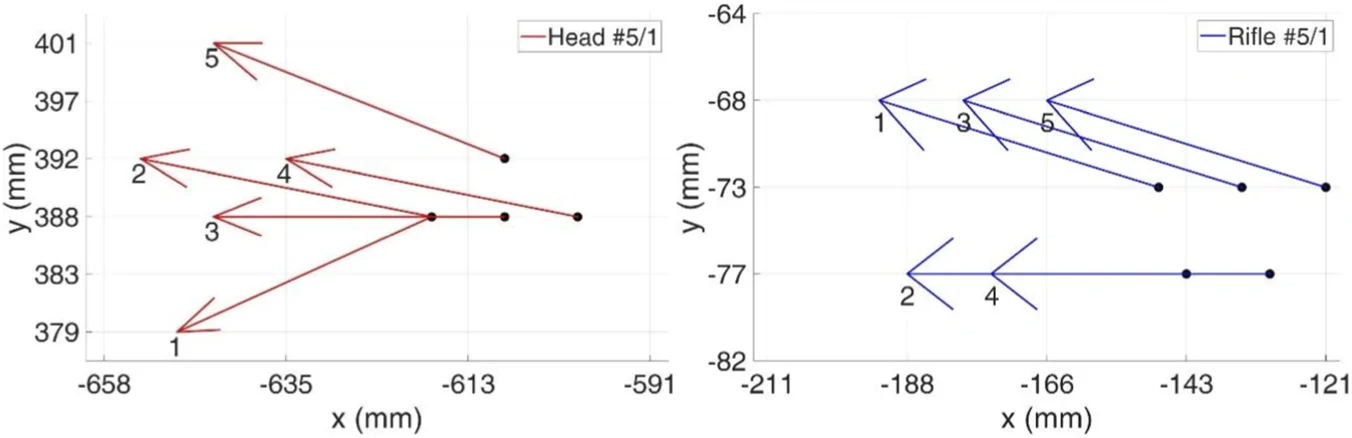
Head (left) and rifle (right) targets displacement for trial 5/1.

**FIGURE 9:**
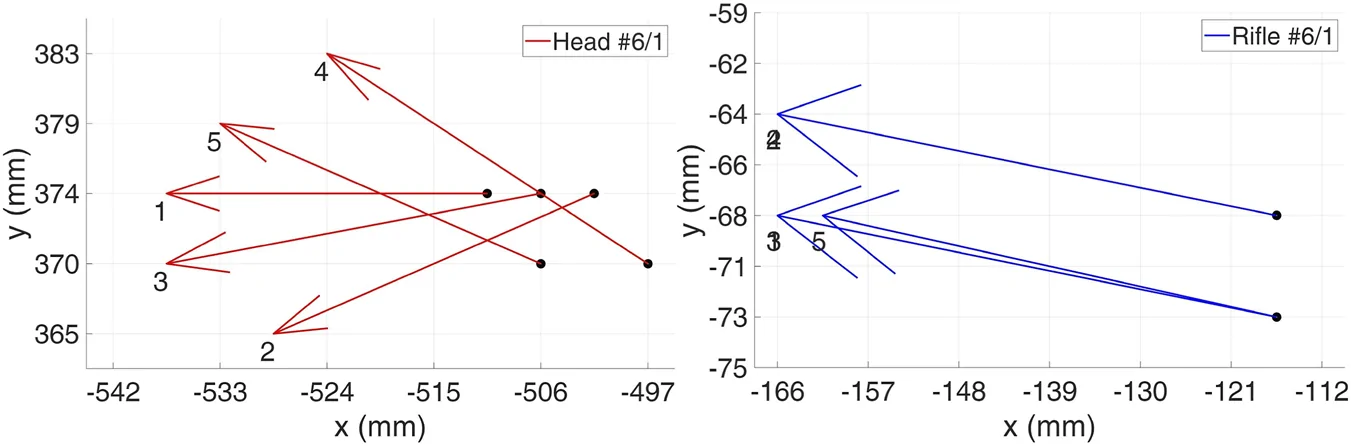
Head (left) and rifle (right) targets displacement for trial 6/1.

**FIGURE 10:**
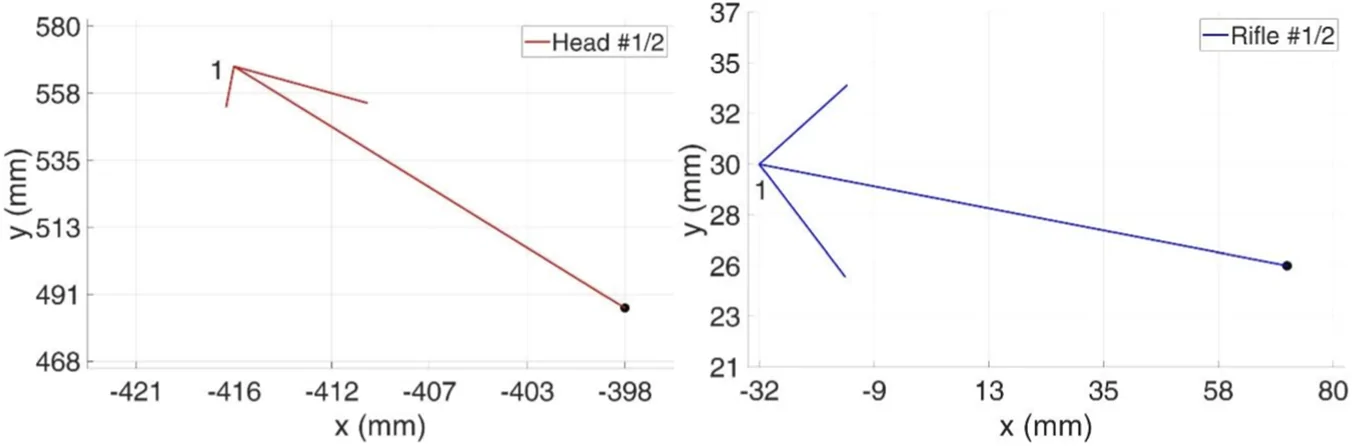
Head (left) and rifle (right) targets displacement for trial 2/1.

**FIGURE 11:**
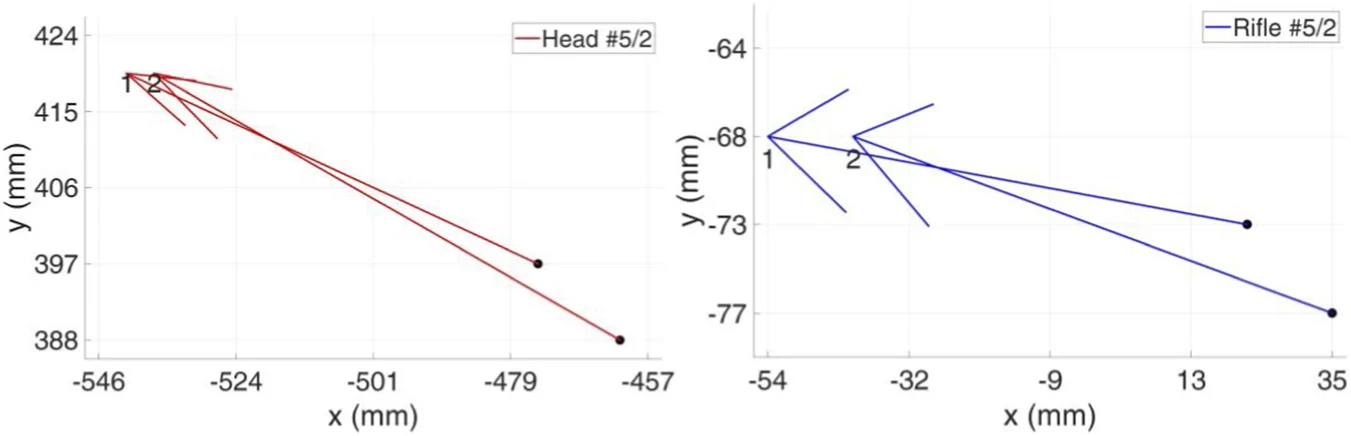
Head (left) and rifle (right) targets displacement for trial 5/2.

In Figures 4–11, the black dot represents the target centre, and the vector extends from this point to the position of maximum displacement recorded during firing. The coordinate system is defined with its origin at the target centre during the first rifle firing for each participant.

The results for maximum rifle displacement, maximum head displacement, and maximum head acceleration across all participants are presented in Tables 2–4, respectively. If a value in a particular cell is missing, the corresponding measurement was corrupted and could not be included in the analysis (for calculating average).

**TABLE 2:** Rifle displacement.

Participant (#)	Muzzle brake (yes/No)	Trials	Rifle maximum displacement (mm)					
			1	2	3	4	5	Ø
1	Yes	1/1	​	45.61	44.95	13.42	41.24	36.30
	No	1/2	102.97	​	​	​	​	102.97
2	Yes	2/1	50.01	49.40	45.61	46.70	46.70	47.68
3	Yes	3/1	50.01	53.86	49.40	44.95	50.01	49.64
4	Yes	4/1	68.42	67.68	62.78	67.24	72.12	67.65
5	Yes	5/1	44.95	44.73	44.95	44.73	44.95	44.86
	No	5/2	76.17	76.56	​	​	​	76.36
6	Yes	6/1	49.40	49.40	49.40	49.40	44.95	48.51

**TABLE 3:** Head displacement.

Participant (#)	Muzzle brake (yes/No)	Trials	Head maximum displacement (mm)					
			1	2	3	4	5	Ø
1	Yes	1/1	​	30.00	30.00	13.42	30.00	25.86
	No	1/2	82.47	​	​	​	​	82.47
2	Yes	2/1	32.56	32.56	28.29	27.21	31.63	30.45
3	Yes	3/1	41.24	36.06	36.88	36.06	41.24	38.29
4	Yes	4/1	49.40	44.73	41.24	42.43	44.95	44.55
5	Yes	5/1	32.56	36.06	35.78	36.06	36.88	35.47
	No	5/2	70.72	82.23	​	​	​	76.47
6	Yes	6/1	26.84	28.29	31.63	30.00	28.29	29.01

**TABLE 4:** Head acceleration.

Participant (#)	Muzzle brake (yes/No)	Trials	Head maximum acceleration (g)					
			1	2	3	4	5	Ø
1	Yes	1/1	3.78	3.18	​	3.97	3.92	3.71
	No	1/2	8.71	​	​	​	​	8.71
2	Yes	2/1	9.15	9.17	6.79	4.92	8.45	7.70
3	Yes	3/1	3.49	3.35	4.04	9.30	10.78	6.19
4	Yes	4/1	7.50	8.51	6.64	9.13	9.57	8.27
5	Yes	5/1	12.88	7.06	5.42	7.78	4.45	7.52
	No	5/2	5.47	4.73	​	​	​	5.10
6	Yes	6/1	10.14	8.07	8.04	9.69	7.16	8.62

Additionally, for clarity, Table 5 presents the average values of maximum rifle displacement, maximum head displacement and maximum head acceleration across all participants. Both additional trials without the muzzle brake are presented at the end.

**TABLE 5:** Average maximum values across all firing trials per participant.

Participant (#)	Muzzle brake (yes/No)	Trials	Rifle	Head	
			Displacement (mm)	Displacement (mm)	Acceleration (g)
1	Yes	1/1	36.30	25.86	3.71
2		2/1	47.68	30.45	7.70
3		3/1	49.64	38.29	6.19
4		4/1	67.65	44.55	8.27
5		5/1	44.86	35.47	7.52
6		6/1	48.51	29.01	8.62
1	No	1/2	102.97	82.47	8.71
5		5/2	76.36	76.47	5.10

## Discussion

This pilot study investigated rifle and head kinematics during firing using automated motion tracking to quantify maximum displacement and head acceleration. Although the comparison between firing with and without a muzzle brake was limited to two participants, the observations suggest that the use of a muzzle brake may reduce the translational recoil motion of both the rifle and the shooter’s head. However, due to the limited number of trials without the muzzle break, such comparison is descriptive and exploratory rather than inferential.

More importantly, the study demonstrates the feasibility of combining high-speed video tracking with inertial measurements to objectively quantify recoil biomechanics under realistic shooting conditions. The observed inter-individual variability further highlights the potential influence of anthropometric and biomechanical factors on recoil response, providing a basis for future investigations aimed at optimizing firearm ergonomics, shooter safety, and equipment design.

Analysis of the displacement vectors demonstrated that head motion generally followed the recoil-induced displacement of the rifle, indicating mechanical coupling between the weapon and the shooter. However, head displacement vectors exhibited greater directional variability than rifle displacement vectors. While rifle displacement was generally concentrated in a consistent direction, head displacement showed a larger spread of vector orientations between trials and participants. This finding suggests that recoil transmission through the shooter is influenced by individual posture, body configuration, head-rifle contact, and neck stiffness, resulting in more variable head motion patterns compared with the rifle itself.

Inter-individual variability was evident across all measures, which is likely influenced by differences in anthropometry, including body mass, stature, and head-neck geometry, all of which may affect recoil transmission and absorption strategies. A potential gender-related trend was observed, with female participants tending to exhibit higher displacement values compared with male participants under comparable conditions. This may reflect differences in anthropometric proportions and neck musculature, which can influence head stabilization and recoil damping capacity. However, due to the limited sample size, these observations should be interpreted cautiously and considered exploratory.

Clear differences were observed between conditions with and without a muzzle brake. With the muzzle brake installed, lower values of rifle and head displacement were observed across participants, with rifle displacement ranging from 36.30 to 67.65 mm and head displacement from 25.86 to 44.55 mm. Peak head acceleration ranged from 3.71 to 8.62 g. In contrast, trials without the muzzle brake exhibited substantially higher kinematic responses, with rifle displacement increasing to 76.36–102.97 mm and head displacement to 76.47–82.47 mm. Head acceleration also increased, reaching up to 8.71 g. In the limited observations available without the muzzle brake, the use of a muzzle brake was associated with reduced rifle and head displacement. However, the magnitude and direction of the kinematic response varied between individuals, suggesting that anthropometric factors may influence recoil biomechanics in addition to equipment configuration. Although differences between male and female participants were observed, the sample size was too small to draw any conclusions regarding gender-related effects.

This study has several limitations that should be considered when interpreting the results. First, the sample size was relatively small, which limits the statistical power and generalisability of the findings. In particular, the number of participants does not allow for robust inference regarding inter-individual variability or subgroup comparisons. The participant group was not fully balanced also in terms of anthropometric characteristics and gender, which further restricts the ability to draw firm conclusions about the influence of anthropometry on recoil-related kinematics. Any observed differences between individuals should therefore be interpreted as exploratory rather than definitive. Further, only a limited number of firing trials were available for each participant, and additional trials without the muzzle break were performed only for selected individuals. This uneven distribution of conditions may introduce bias when comparing recoil responses across configurations. Finally, the analysis was based on external kinematic measures derived from automated tracking, which may be affected by marker placement accuracy, soft tissue artefacts, and potential tracking noise. No dedicated validation experiment was performed to quantitatively assess the tracking error. However, the use of high-contrast circular markers and a template-based tracking approach minimized the risk of tracking failures and ensured consistent identification of the marker centres throughout the recordings. The lack of a quantitative uncertainty assessment represents a limitation of the present study and should be considered when interpreting the displacement results.

Potential measurement artefacts should be also acknowledged for acceleration. Because the accelerometer was mounted on the forehead rather than directly to the skull, the recorded accelerations may have been influenced by deformation of the intervening soft tissues, representing an inherent limitation of non-invasive measurements. Magnetic resonance imaging studies have reported a forehead soft-tissue thickness of approximately 5.0–5.4 mm (Cakmakoglu et al., 2025), while the Young’s modulus of facial soft tissue has been reported as 17.3 ± 4.1 kPa (Luboz et al., 2014). These findings indicate that the intervening tissue exhibits measurable compliance and therefore may attenuate the acceleration transmitted from the skull to the externally mounted sensor. Although the mounting strap was tightened to the maximum level tolerated by each participant to minimize relative sensor motion, some residual strap movement cannot be completely excluded. In addition, cable-induced loading was minimized by incorporating a strain-relief loop immediately proximal to the accelerometer connector, thereby reducing the transmission of cable motion to the sensor during firing.

The fixed order of the experimental conditions represents an additional limitation of the study. As the muzzle brake condition was performed before the condition without the muzzle break, potential order effects, including anticipation, learning, fatigue, pain, or postural adaptation, cannot be excluded. Furthermore, only two participants completed the condition without the muzzle break for safety reasons; therefore, findings from this condition should be considered exploratory and interpreted with caution.

## Conclusion

This study investigated rifle and head kinematics during firing under conditions with and without a muzzle brake, using automated motion tracking to quantify maximum displacement and head acceleration across participants. All participants completed firing with the muzzle brake, whereas only two participants performed a limited number of additional firing trials without the muzzle brake. These preliminary observations suggest that the use of a muzzle brake may reduce translational displacement of both the rifle and the shooter’s head, with the most pronounced effect observed in rifle motion and the resulting head displacement. Given the limited and unbalanced comparison, these findings should be interpreted as exploratory and require confirmation in larger, balanced studies.

Peak head acceleration showed a less consistent response across participants, suggesting that recoil mitigation primarily affects displacement-related kinematics rather than peak inertial loading. Considerable inter-individual variability was observed, indicating that individual biomechanical and anthropometric characteristics likely influence recoil transmission between the rifle and the shooter.

Although exploratory observations suggested possible gender- and anthropometry-related differences in response to recoil, the limited sample size prevents definitive conclusions. Overall, the findings indicate that muzzle brakes are effective in reducing recoil-induced motion, while individual factors may play an important role in modulating the biomechanical response to firing.

The present findings provide a foundation for ongoing investigations aimed at evaluating the relationship between recoil-induced head kinematics and potential mild traumatic brain injury (mTBI) risk, including the identification of biomechanical factors that may contribute to head loading during high-energy firearm recoil.

## Data Availability

The raw data supporting the conclusions of this article will be made available by the authors, without undue reservation.
